# Supplementary material for: Activity of Estafietin and Analogues on Trypanosoma cruzi and Leishmania braziliensis
Source: Molecules. 2019 Mar 28;24(7):1209. doi: 10.3390/molecules24071209 (PMC6479983; doi:10.3390/molecules24071209)
Supplement: Supplementary file 1 [file molecules-24-01209-s001.pdf]

Supplementary Materials

Activity of estafietin and analogues on *Trypanosoma cruzi* and *Leishmania braziliensis*

Valeria P. Sülsen[1,2,ψ,*], Emilio F. Lizarraga[3,ψ], Orlando G. Elso[1,2], Natacha Cerny[4], Andrés Sanchez Alberti[5], Augusto E. Bivona[5], Emilio L. Malchiodi[5], Silvia I. Cazorla[5,6], and César A. N. Catalán[7,*]

[1] CONICET – Universidad de Buenos Aires. Instituto de Química y Metabolismo del Fármaco - CONICET (IQUIMEFA), Junín 956 2°P (1113). Buenos Aires, Argentina; vsulsen@ffyb.uba.ar (VPS), orlandoelso@hotmail.com (OGE).

[2] Universidad de Buenos Aires, Cátedra de Farmacognosia, Facultad de Farmacia y Bioquímica, Junín 956 2°P (1113). Buenos Aires, Argentina.

[3] Instituto de Fisiología Animal, Fundación Miguel Lillo and Facultad de Ciencias Naturales e Instituto Miguel Lillo, Universidad Nacional de Tucumán, Tucumán, Argentina; eflizarraga@lillo.org.ar (EFL)

[4] CONICET - Universidad Nacional de Luján. Instituto de Ecología y Desarrollo Sustentable (INEDES). Ruta 5 y Avenida Constitución - (6700). Luján, Argentina; natachacerny@gmail.com (NC).

[5] Universidad de Buenos Aires, Facultad de Farmacia y Bioquímica, Cátedra de Inmunología. Junín 956 2°P (1113). Buenos Aires, Argentina. Instituto de Estudios de la Inmunidad Humoral (IDEHU), UBA-CONICET. Junín 956 4°P (1113), Buenos Aires, Argentina; CONICET- Universidad de Buenos Aires. Instituto de Microbiología y Parasitología Médica - CONICET (IMPaM), Facultad de Medicina. Paraguay 2155. Piso 13, Buenos Aires, Argentina; augustobivona@gmail.com (AEB), andres.sanchez.alberti@gmail.com (ASA), emalchio@ffyb.uba.ar (ELM).

[6] CONICET – Centro de Referencia para Lactobacilos (CERELA). Batalla de Chacabuco 145. San Miguel de Tucumán, Argentina; silcazorla@yahoo.es (SIC).

[7] CONICET – Universidad Nacional de Tucumán. Instituto de Química del Noroeste - CONICET (INQUINOA). Ayacucho 471 (T4000INI). San Miguel de Tucumán, Argentina; ccatalan@fbqf.unt.edu.ar, cancatalan@gmail.com (CANC).

* Correspondence: vsulsen@ffyb.uba.ar (VPS); ccatalan@fbqf.unt.edu.ar, cancatalan@gmail.com (CANC).

ψ These authors contributed equally to this work.

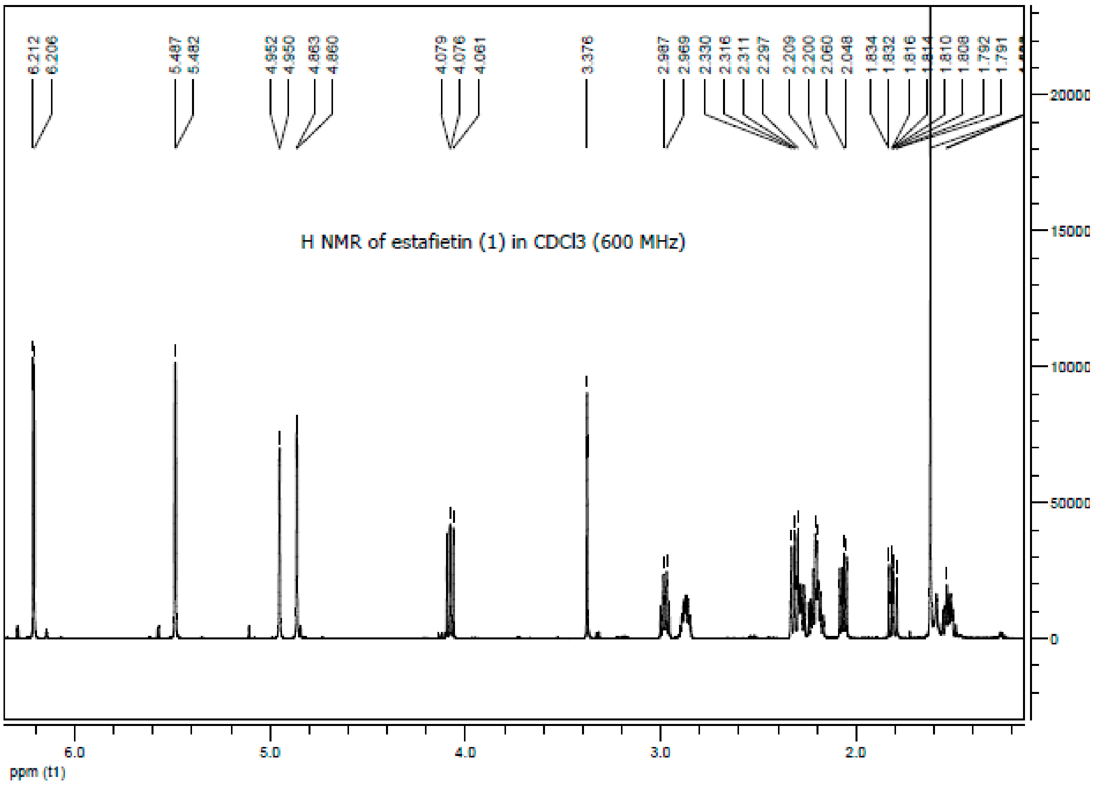

Figure S1. ^{1}H-NMR of estafietin (**1**)

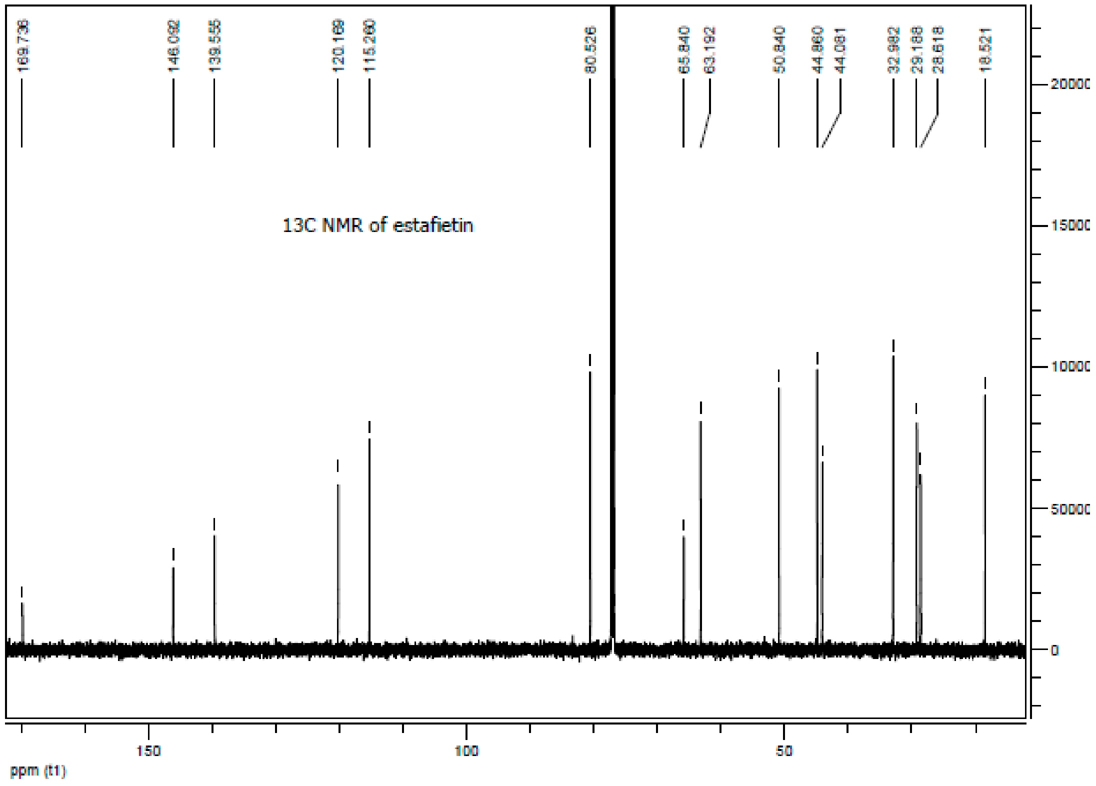

Figure S2. ^{13}C-NMR of estafietin (**1**)

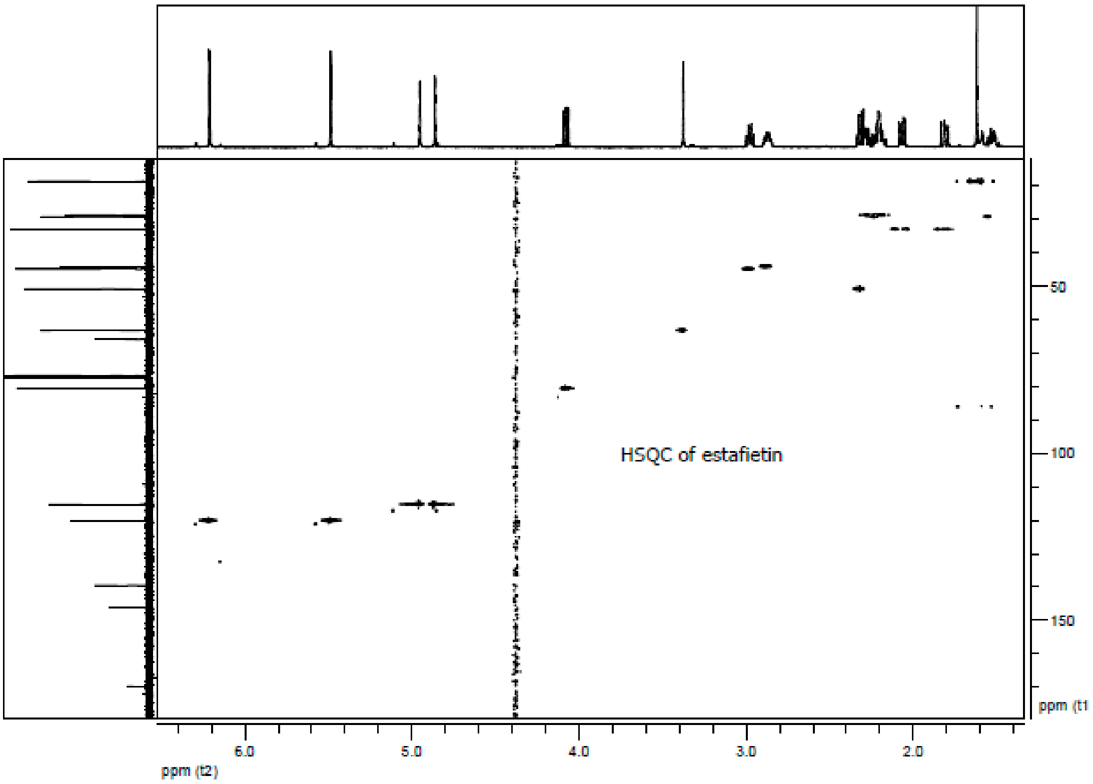

Figure S3. HSQC of estafietin (**1**)

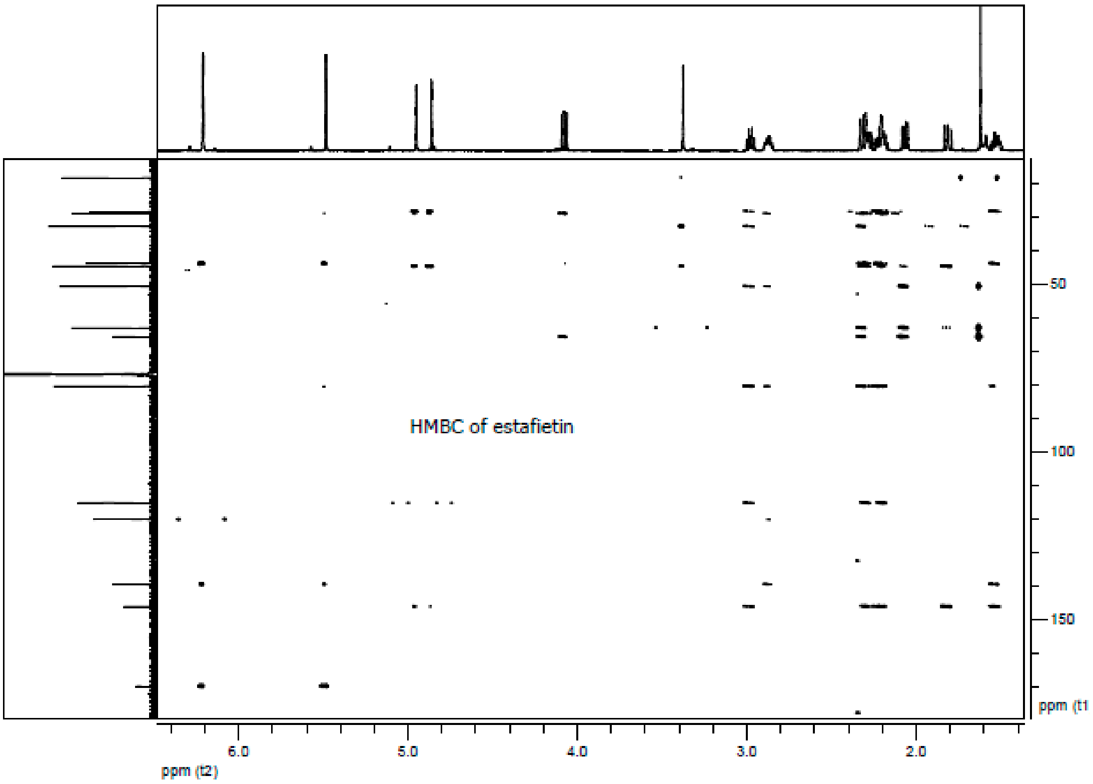

Figure S4. HMBC of estafietin (**1**)

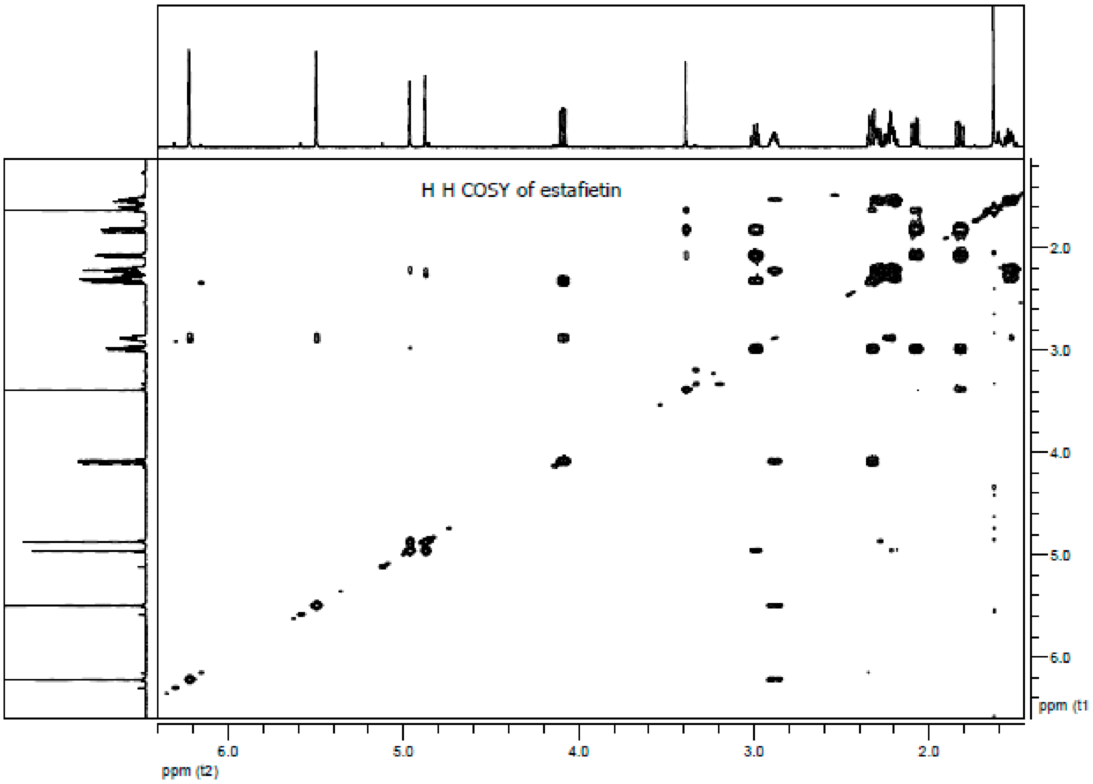

Figure S5. H H COSY of estafietin (**1**)

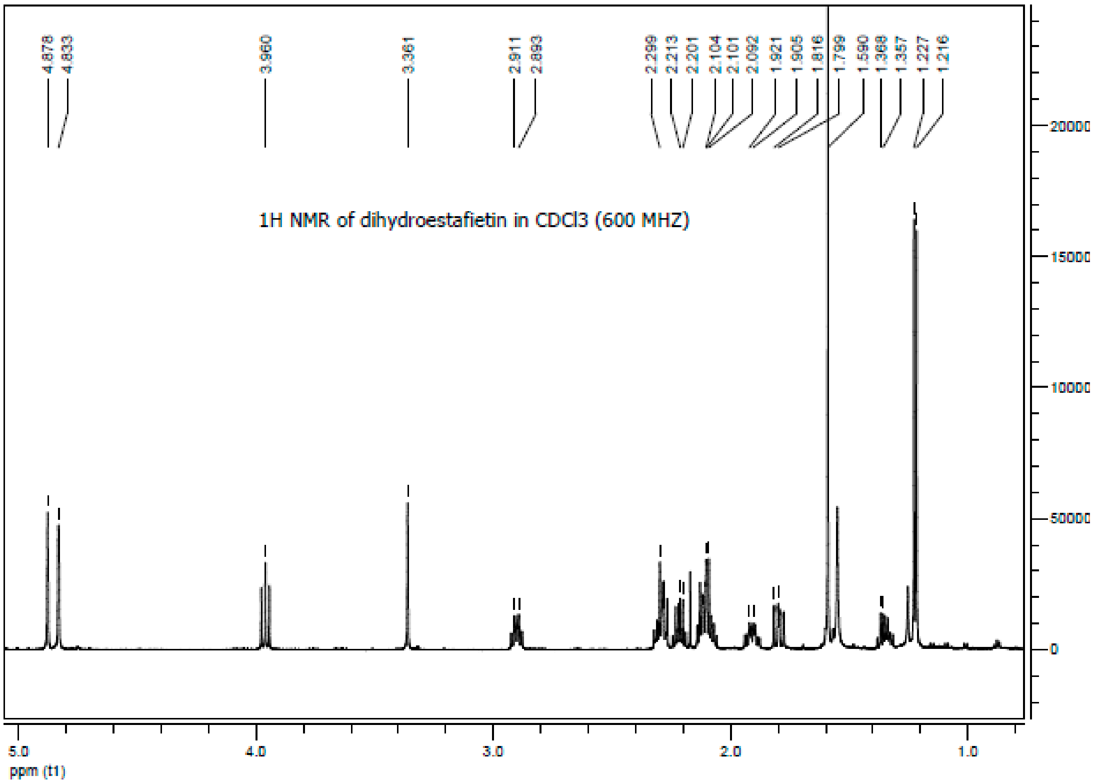

Figure S6. ^{1}H-NMR of 11βH,13-dihydroestafietin (**2**)

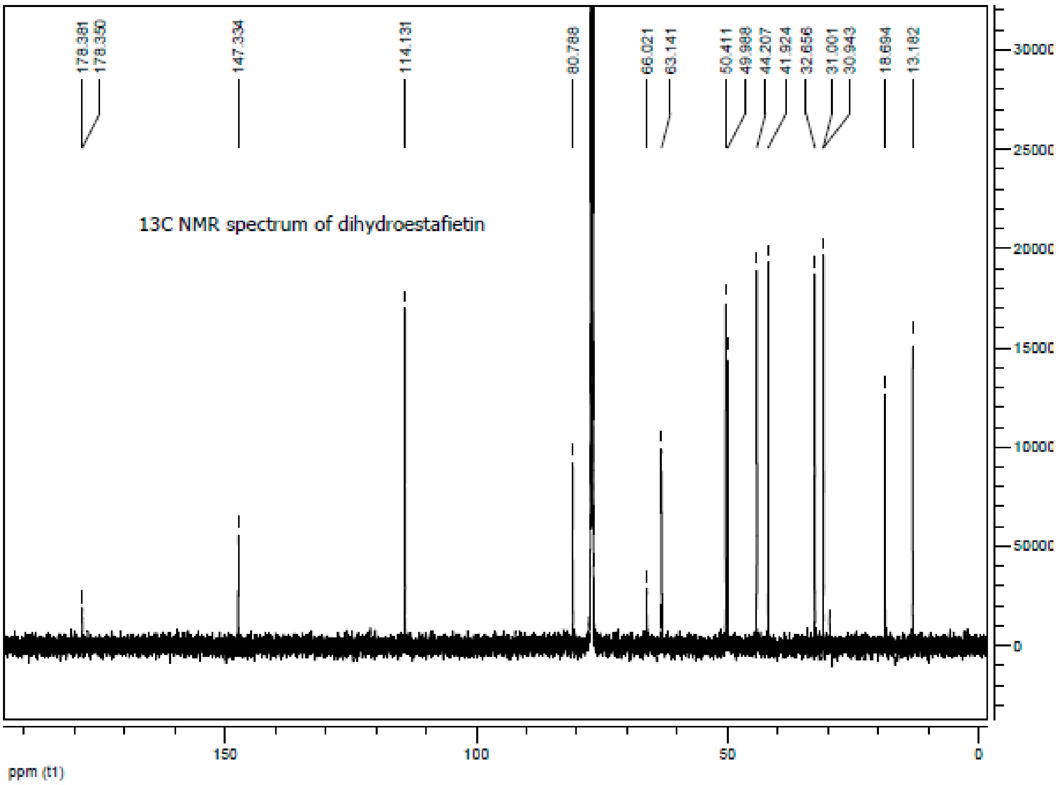

Figure S7. ^{13}C-NMR of 11βH,13-dihydroestafietin (**2**)

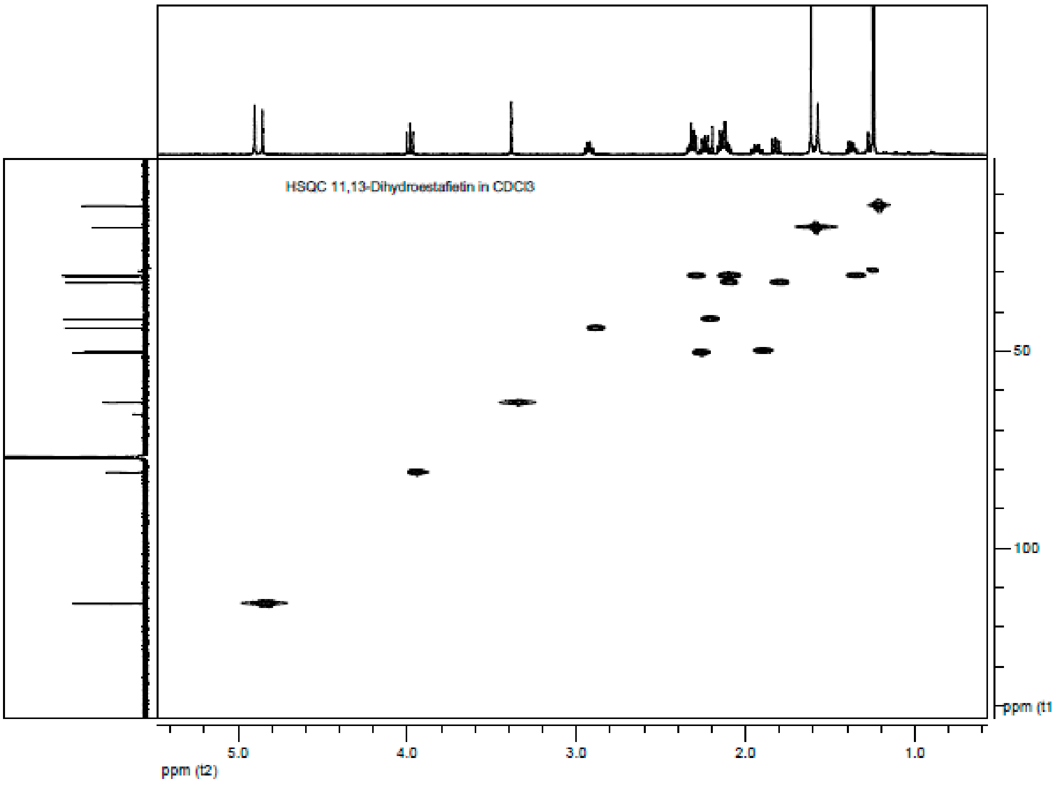

Figure S8. HSQC of 11βH,13-dihydroestafietin (**2**)

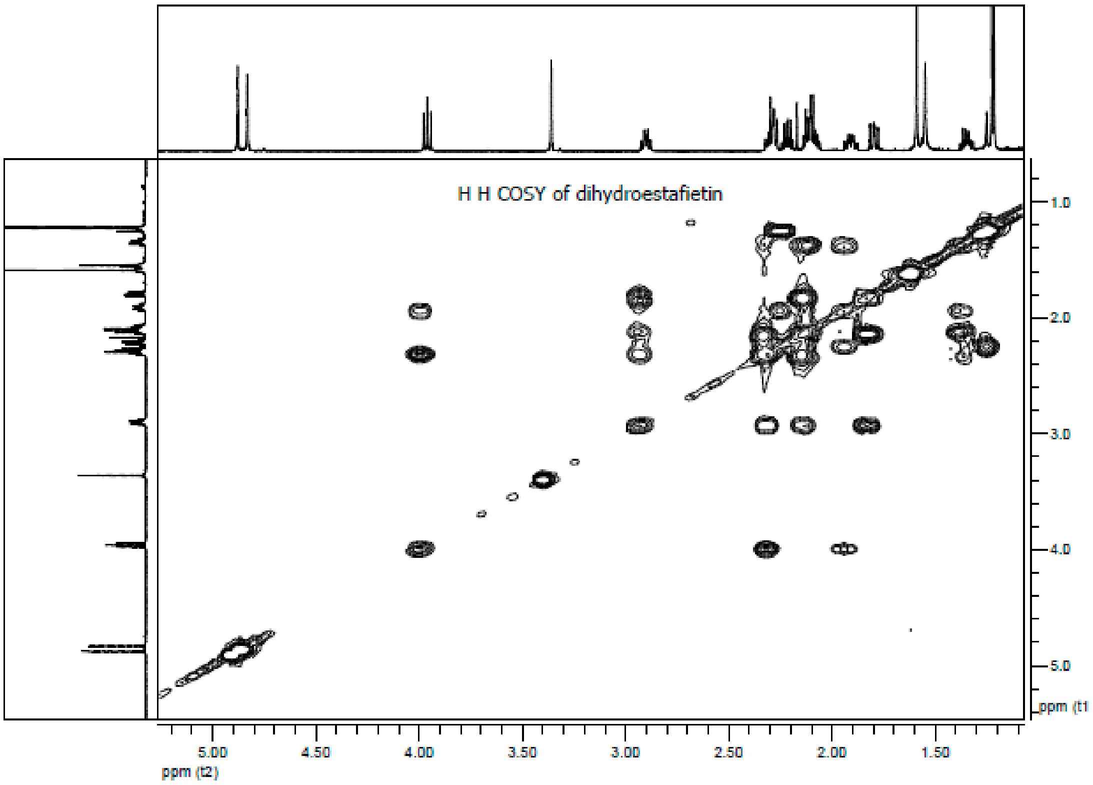

Figure S9. H H COSY of 11βH,13-dihydroestafietin (**2**)

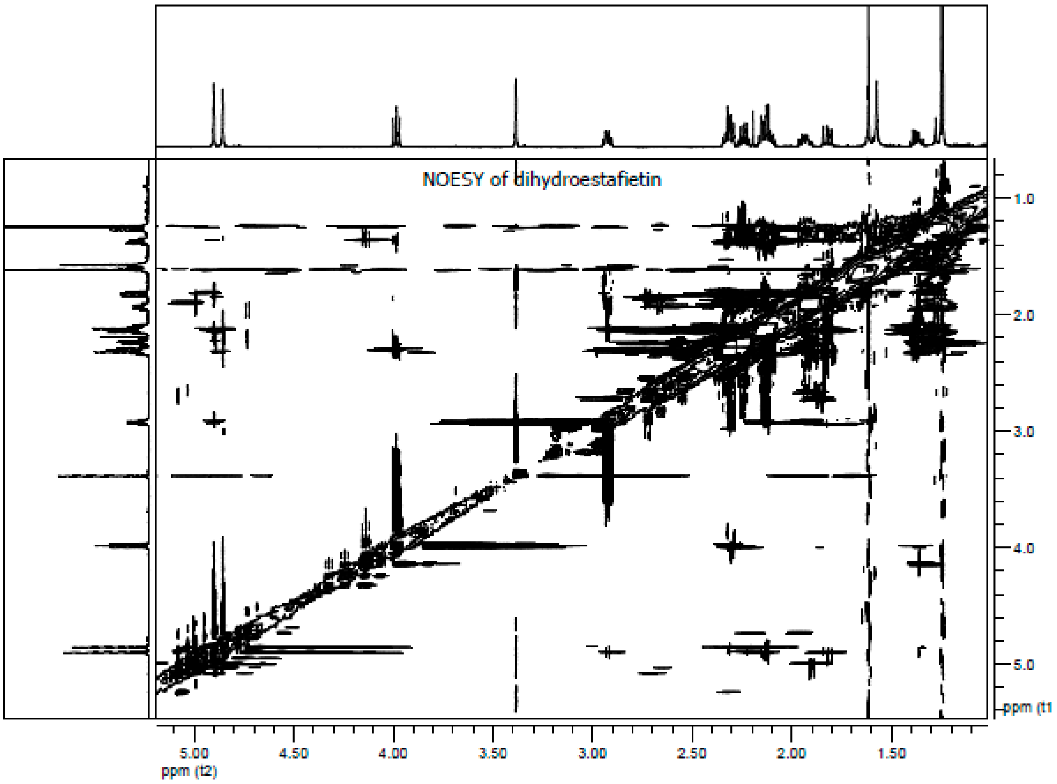

Figure S10. NOESY of 11βH,13-dihydroestafietin (**2**)

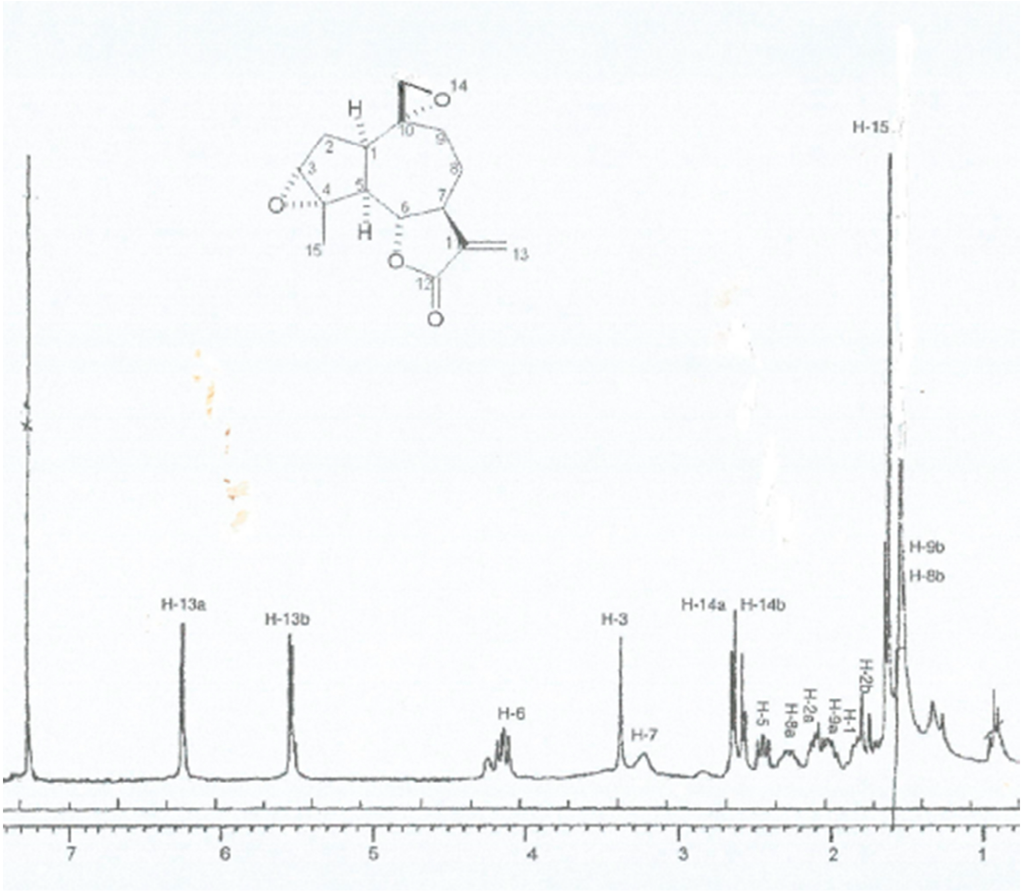

Figure S11. ^{1}H-NMR of α-epoxyestafietin (**3a**)

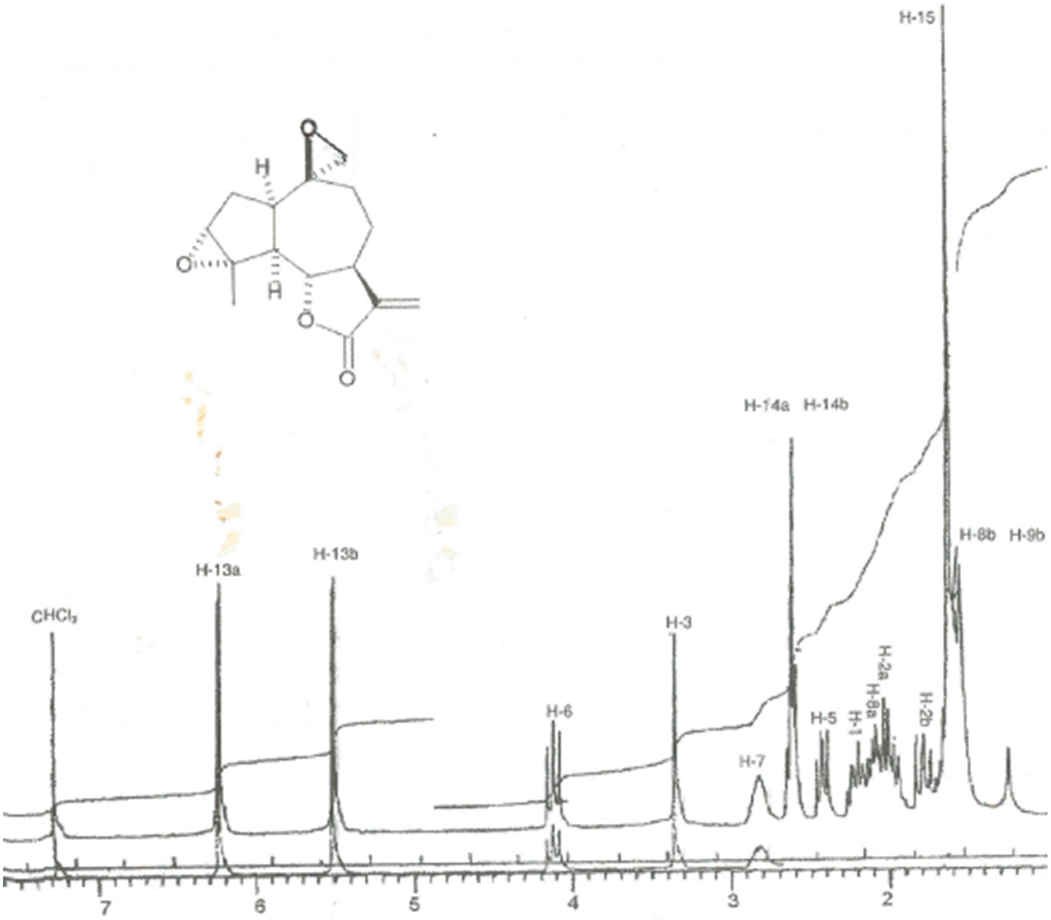

Figure S12. ^{1}H-NMR of β-epoxyestafietin (**3b**)

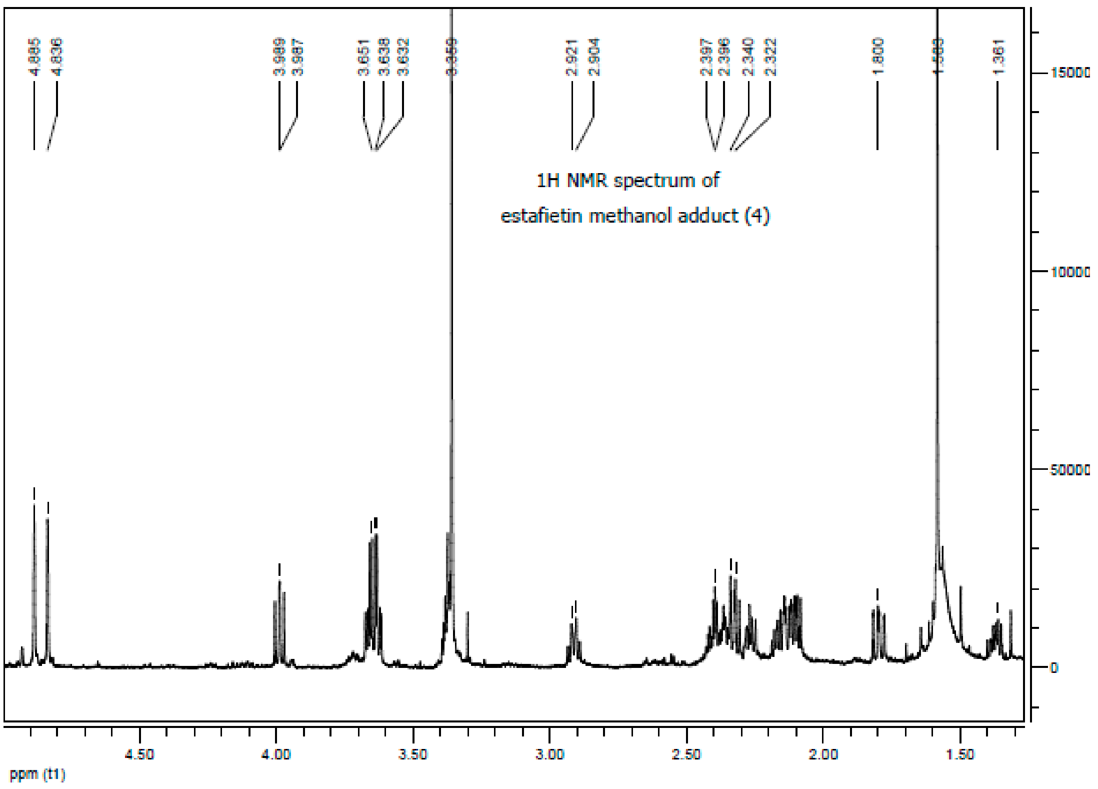

Figure S13. ^{1}H-NMR of 11βH,13-methoxyestafietin (**4**)

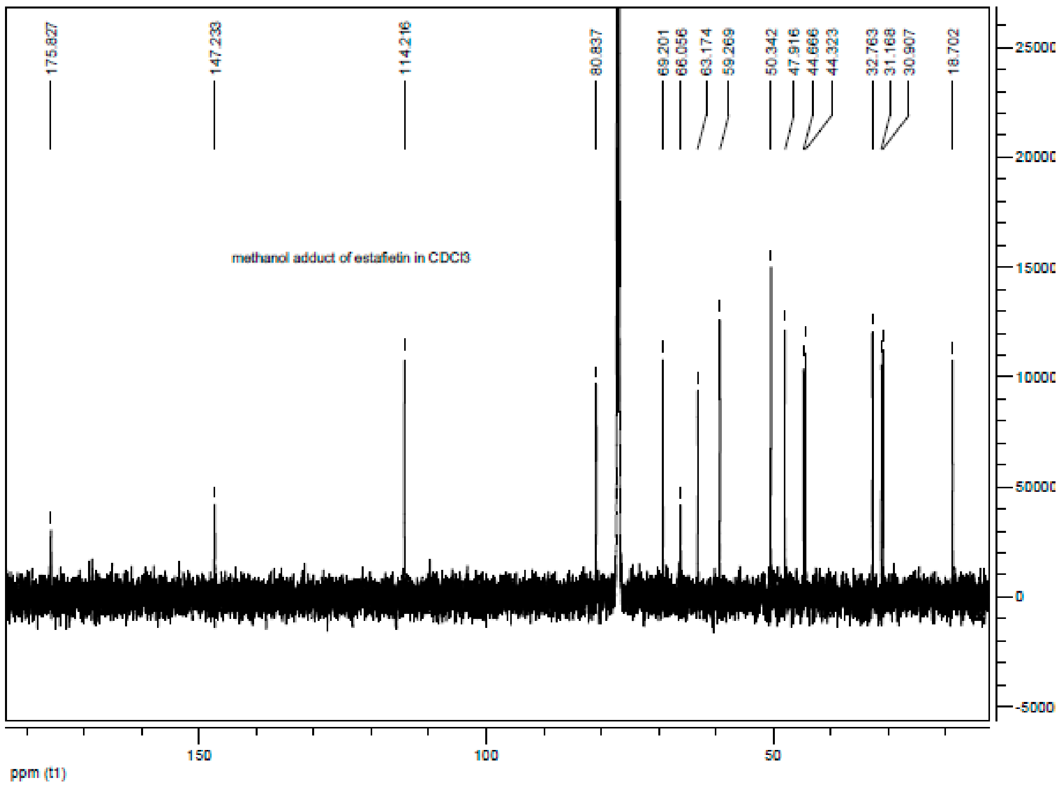

Figure S14. ^{13}C-NMR of 11βH,13-methoxyestafietin (**4**)

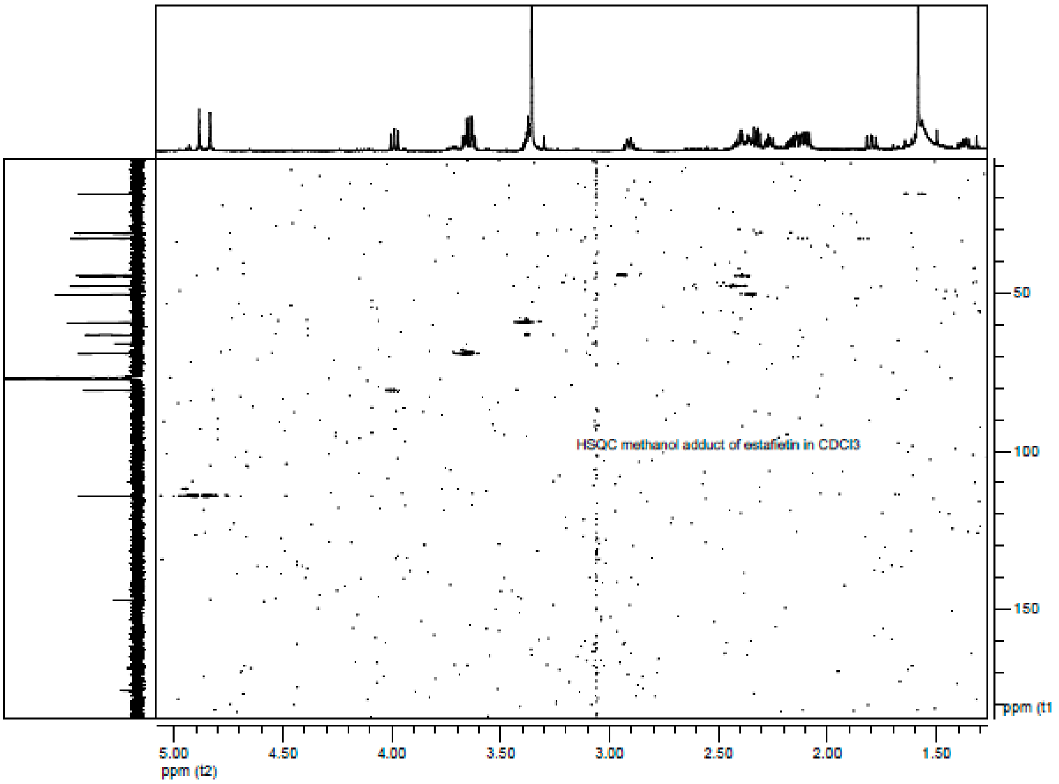

Figure S15. HSQC of 11βH,13-methoxyestafietin (**4**)

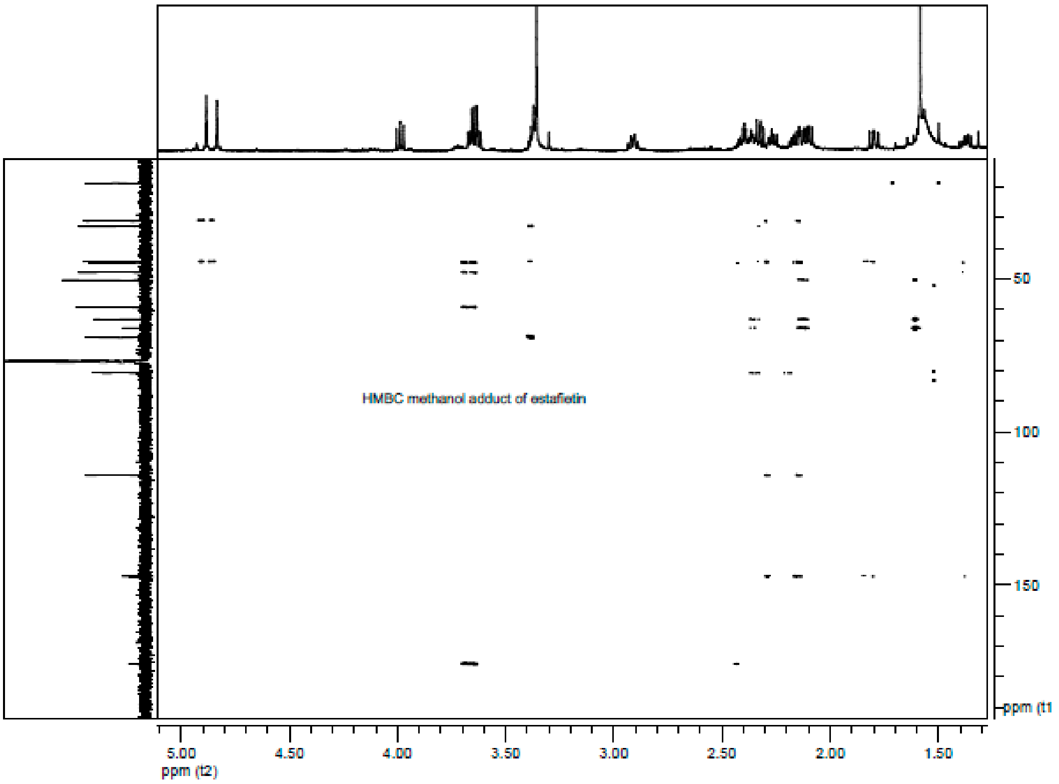

Figure S16. HMBC of 11βH,13-methoxyestafietin (**4**)

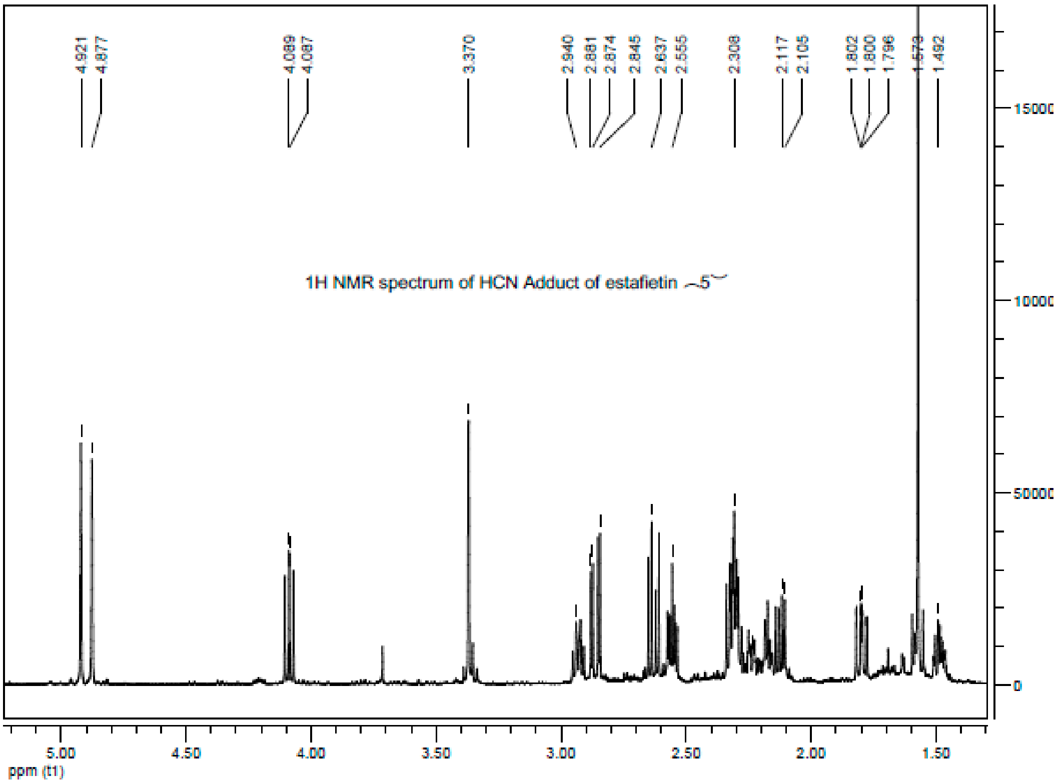

Figure S17. ^{1}H-NMR of 11βH,13-cianoestafietin (**5**)

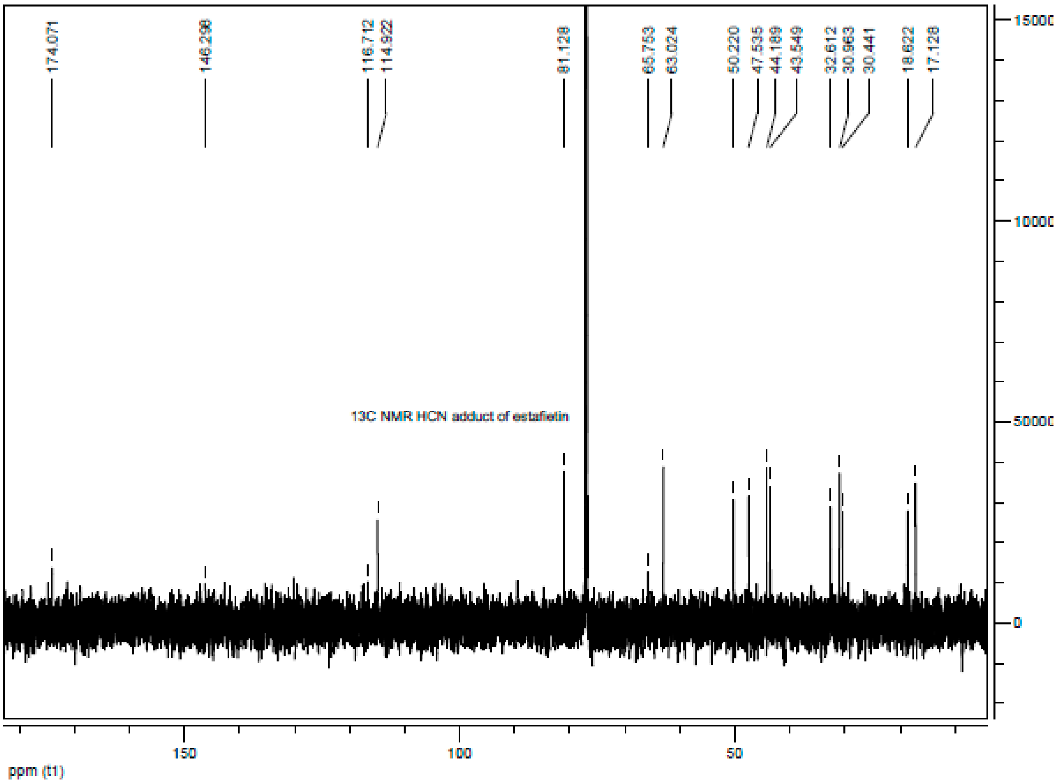

Figure S18. ^{13}C-NMR of 11βH,13-cianoestafietin (**5**)

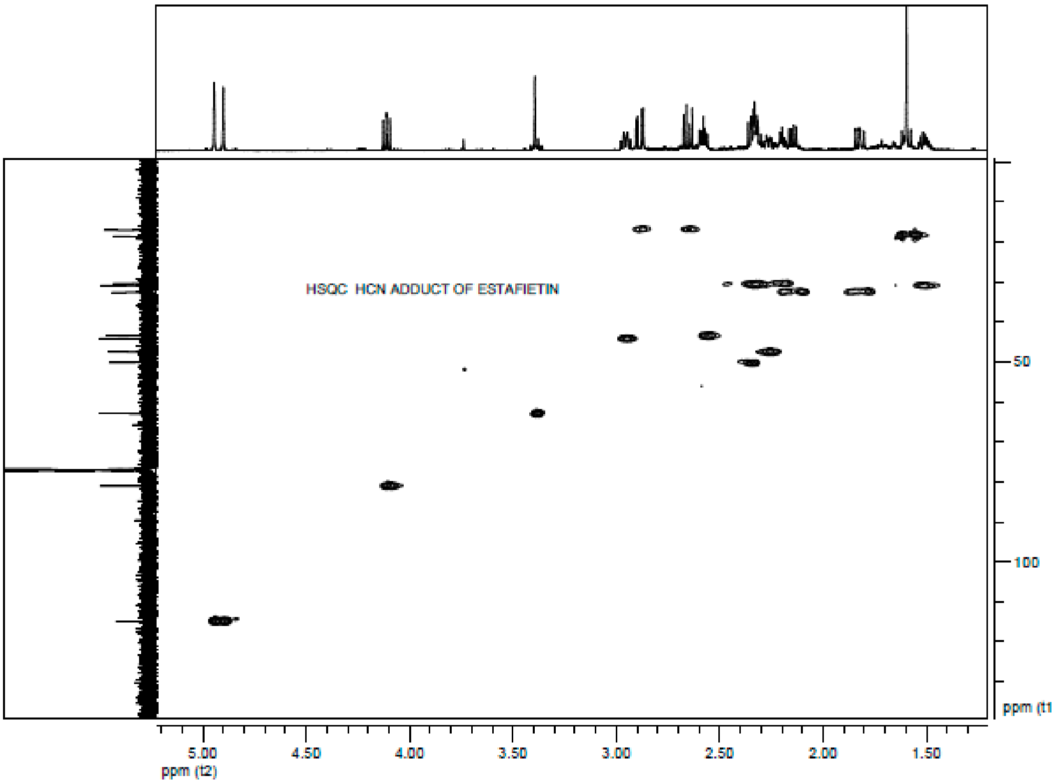

Figure S19. HSQC of 11βH,13-cianoestafietin (**5**)

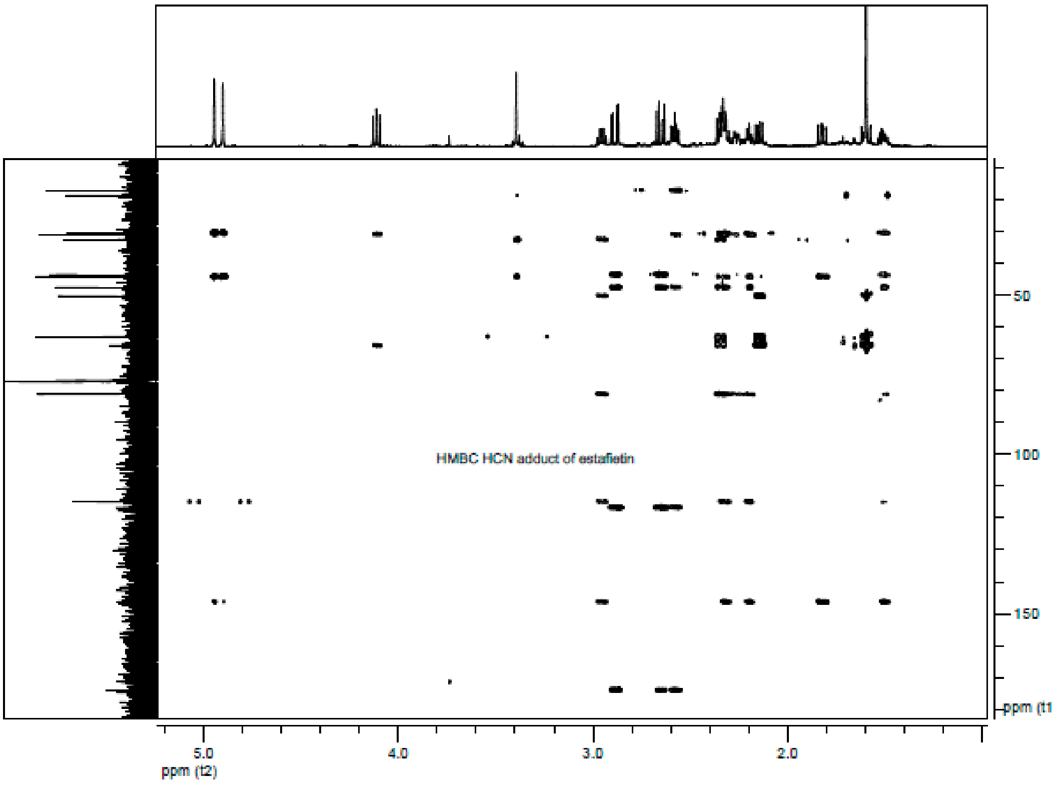

Figure S20. HMBC of 11βH,13-cianoestafietin (**5**)

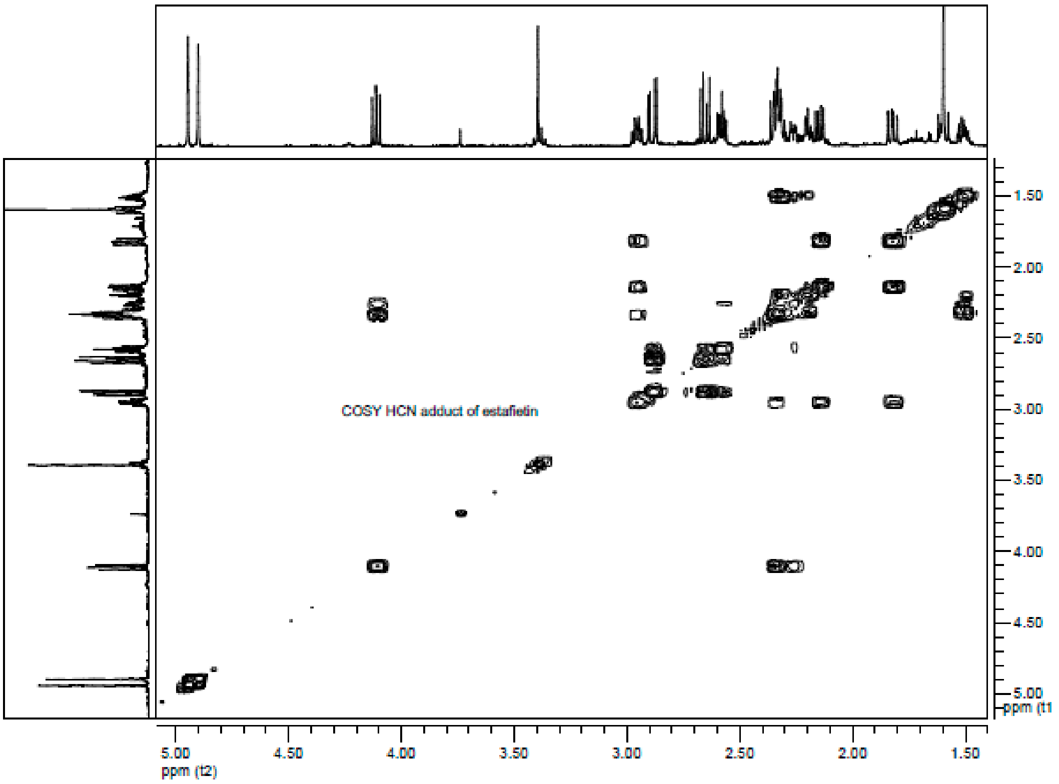

Figure S21. H H COSY of 11βH,13-cianoestafietin (**5**)

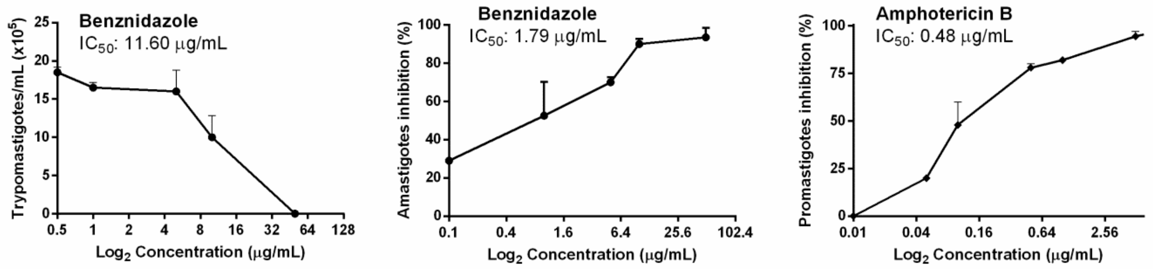

Figure S22. Activity of the reference drugs benznidazol and amphotericin B determined by *in vitro* assays against trypomastigotes and amastigotes of *T. cruzi* and promastigotes of *L. braziliensis*, respectively.
